# The role of a digital twin in supporting criminal investigations - a case report about a possible abuse

**DOI:** 10.1007/s12024-024-00857-w

**Published:** 2024-07-18

**Authors:** Sven Becker, Tim Hanjo Fritzsch, Dirk Labudde

**Affiliations:** 1 Faculty of Applied Computer and Life Sciences, University of Applied Sciences Mittweida, Technikumplatz 17, D-09648 Mittweida, Saxony Germany; 2 weltenbauer. Software Entwicklung GmbH, Frankfurter Str. 5, D-65189 Wiesbaden, Hesse Germany

**Keywords:** Forensics, Legal medicine, 3D reconstruction, Photogrammetry, Structural light, Object measurement

## Abstract

As part of a comprehensive analysis, this case report presents a possible case of child maltreatment that can serve as a basis for forensic and medical examiner investigations. This case concerns the death of an infant who was approximately two months old. During a routine examination by the pediatrician at the end of May 2021, the child was found to have a normal head circumference of 31 cm. No other abnormalities were noted. On June 19, 2021, the child died, and an autopsy revealed a head circumference of 44 cm and a subdural hematoma as the cause of death. Questions arose as to who might have abused the child and when. The only evidence was a low-quality cell phone video taken by the child’s parents on June 13, 2021, six days before the child’s death, in which the child could be seen lying on a pillow. It was necessary to determine whether the child in this video already had an unnatural head circumference. This study presents a novel workflow that demonstrates how to analyze and deal with low quality video to answer questions like the above. The workflow demonstrates the creation of 3D scenes from digital image and video material. These 3D scenes can be used for object measurement and to support forensic and medical investigations. In the present case, where only low quality smartphone images were available, the presented workflow was used to create a 3D scene of the child lying on the pillow. In this 3D scene, it was possible to determine the child’s head circumference. These measurements support the findings of the medical examiner (dated June 24, 2021) and confirm the suspicion that possible child abuse had already taken place on June 13, 2021. The innovative approach makes it possible to identify evidence of possible abuse based on a specific point in time, in this case the child’s private footage. It also demonstrates the potential of 3D scene reconstruction in complex forensic and medical scenarios.

## Introduction

**Fig. 1:**
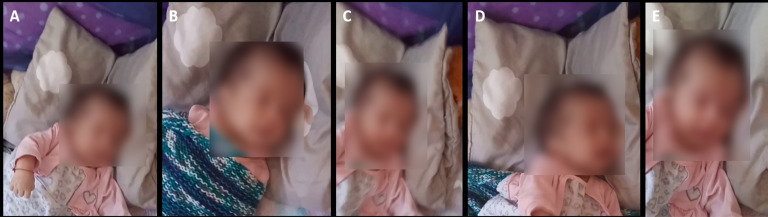
Representation of the child in the private video recordings made during his lifetime and used for the analysis in this paper

In cases of sudden infant death syndrome, the first priority is to determine whether the cause of death was natural or unnatural. If an unnatural cause of death is associated with possible child maltreatment, there are international guidelines to follow when examining the child or performing an autopsy. For example, guidelines for dealing with possible child maltreatment identified during a normal examination of the child, such as those from the World Health Organization [1]. Guidelines for a post-mortem examination include examination of the eyes and eye sockets, as well as skeletal changes [2–5]. If the child has been abused and it has resulted in death, it is important to find out not only who abused the child, but also when. Often there is no information about the possible course of events other than witness statements. However, if there are photographs or video recordings of the child when he or she was alive, they may contain helpful information about abuse that has already occurred. One way to do this would be to record any abnormalities in the child, such as an enlarged head. However, since this is only a two-dimensional representation, these surveys would be subject to considerable error [6]. In order to still be able to make reliable measurements, it is possible to calculate a 3D scene from the photo and video recordings and also to digitize the depicted objects. Methods such as photogrammetry [7–12] and laser scanning [13, 14] are used for this purpose. These are already used in forensics and legal medicine for documentation purposes and for measuring injuries [15, 16]. However, these methods also have disadvantages, such as the non-real scale digitization of a scene using photogrammetry. Reference dimensions and methods like superimposition [17, 18] can be used here to create true-to-scale and true-to-scale 3D scenes in combination. These 3D scenes now make it possible to measure objects within them, in this case the circumference of a child’s head [19–24]. The paper presented here is a case report of possible child abuse. The goal of this paper is to calculate a 3D scene from the underlying video footage of the child and, by measuring the circumference of the child’s head, to provide evidence that the child had already been abused at the time the footage was taken.

## Case report

A forensic examination identified a possible case of child abuse. A routine examination of the child by the attending pediatrician revealed no evidence of abuse. At the end of May 2021, the pediatrician measured the child’s head circumference to be 31 cm. This was within the normal range for the child’s age. Fourteen days after the medical examination, on June 13, 2021, the child’s parents took photographs and a video (evidence video, excerpts shown in Fig. 1) of their child lying on a pillow. The abuse was not initially apparent in these images. Eleven days after the evidence video, on June 24, 2024, a forensic autopsy was performed on the child’s body after his death. The circumference of his head was measured to be 44 cm. The increase in head circumference in such a short period of time seemed to deviate from the norm. During the post-mortem examination, a nonfading lividity was observed on the posterior aspect of the body. The abdominal skin had a greenish tinge. The child’s head was conspicuously large and disproportionate, with the scalp appearing elongated and ballooning. The dart suture was palpable at a distance of approximately 3 cm, while the coronal suture was palpable at a distance of approximately 1.5 cm. Both the anterior and posterior fontanels exhibited bulbous and plate-like characteristics, while the forehead appeared relatively high and markedly bulbous. The facial skull and bony nasal skeleton were found to be stable. Corneas were completely opaque. The eyelids and conjunctivae were pale and poorly vascularized with no evidence of punctate hemorrhages. The external auditory canals were unobstructed, the posterior ear regions were uninjured, and the ears appeared relatively low in relation to the rest of the head. The skin of the neck was uninjured. The thorax was elastic and appeared stable. The abdominal wall showed signs of putrefaction, which had caused it to be distended. A 3 cm x 2 cm skin ulcer was noted in the left axilla. The dorsal aspect of the body was unremarkable. No external signs of malformations or syndromic diseases were observed. The death certificate stated that the cause of death was hypoxia due to aspiration in the context of a developmental disorder in premature birth. However, the autopsy revealed that the cause of death was central paralysis due to chronic progressive subdural hematoma. Subdural hematomas are common in infancy and often result from shaking trauma. They are often associated with retinal hemorrhages and associated injuries such as rib fractures. The child was found to have rib fractures in a typical position that could not be attributed to resuscitation. This suggests a history of violence. The goal was to determine if the child had been abused at the time of the parent’s admission. It is of the utmost importance to obtain an accurate measurement of the child at the time of recording, as this will provide invaluable insight into the dimensions, proportions and structural characteristics of potential injuries. This information can assist in determining the temporal parameters of a potential incident of abuse. Of particular interest in this case was the child’s pillow that was available as evidence. This pillow was used as a size reference between the analog and digital worlds.

## Materials, experimental setup for scans and software applications

### Materials

The evidence video provided has as data format *.media.0 with a resolution of 720 x 1488 px. The length of the evidence video is 10 sec, the memory size is 5.09 MB. The laser scanner used is the 3D structured light handheld scanner "Artec Leo" from Artec 3D. According to the manufacturer, the scanner has a 3D point accuracy up to 0.1 mm, a 3D resolution up to 0.2 mm and a 3D distance accuracy up to 0.1 mm + 0.3 mm/m [25]. The pillow is flat and light gray with white clouds and a white sheep printed on it. The pillow also shows the phrase ”Träum süss” in black letters on both sides.

### Experimental setup for scans

**Fig. 2:**
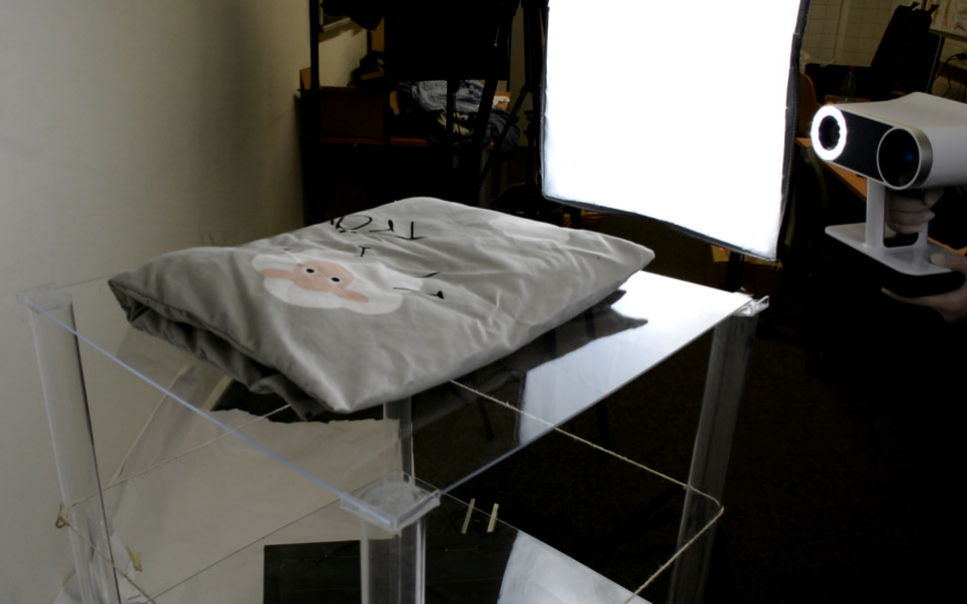
The experimental setup for scanning the pillow with the handheld scanner consists of two studio lights and a fabricated glass table with a tripod mounted on a turntable

For scanning the pillow with the handheld scanner, the experimental setup shown in Fig. 2 consists of two studio lights (MVPower company) for better illumination and a custom-made glass table with tripod (Cullmann company) positioned on a turntable (stageonair, base size 60, diameter 63 cm).

### Software applications

The software VLC (https://www.videolan.org/vlc/index.de.html), version 3.0.8, was used to decompose the underlying evidence video into its individual frames. Meshroom (https://alicevision.org/) is a free, open-source 3D reconstruction software to compute a 3D scene from the extracted frames. Version 2021.1.1.0 was used. The proprietary software Artec Studio (https://www.artec3d.com/de/3d-software/artec-studio), version 17.1.215, was used to scan the underlying pillow. All collected 3D data, pillow and scene, were merged in Blender software (https://www.blender.org/). Blender is an open source software package for modeling, texturing and animation. Version 3.5 was used.

**Fig. 3:**
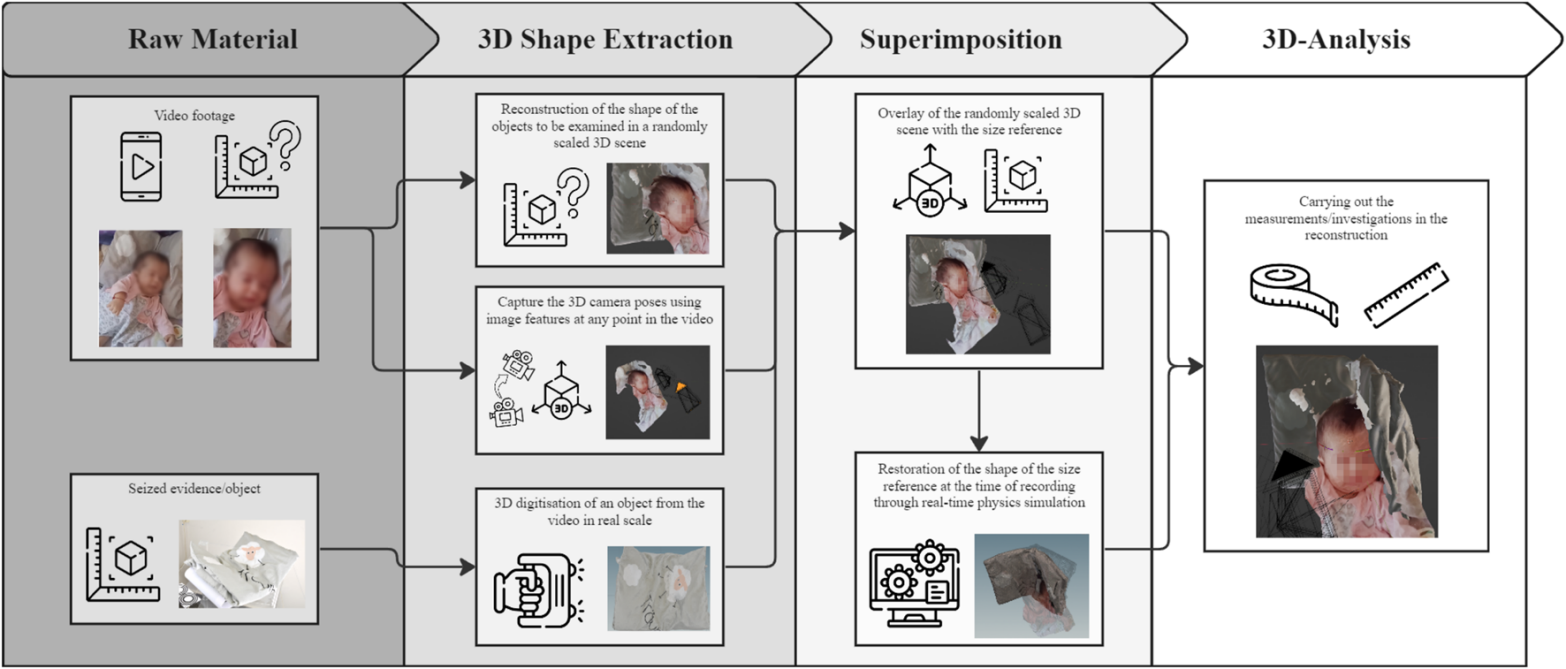
Workflow for crime scene reconstruction using camera motion and a physically present object as a size reference. Structure from Motion is used to derive the shape of the objects in the evidence video from the raw material, such as the video footage, as well as the relative position of the camera to the objects in the scene. The 3D shape extraction of the objects in the raw material is then aligned with the 3D digital copy of the size reference to bring the scene to the real scale. This process is called superimposition. A 3D analysis, such as measurements, can then be made in the virtual scene. The icons used in the image are based on a design by [26]

## Workflow and methods

### Extraction of depth information from the raw material

**Fig. 4:**
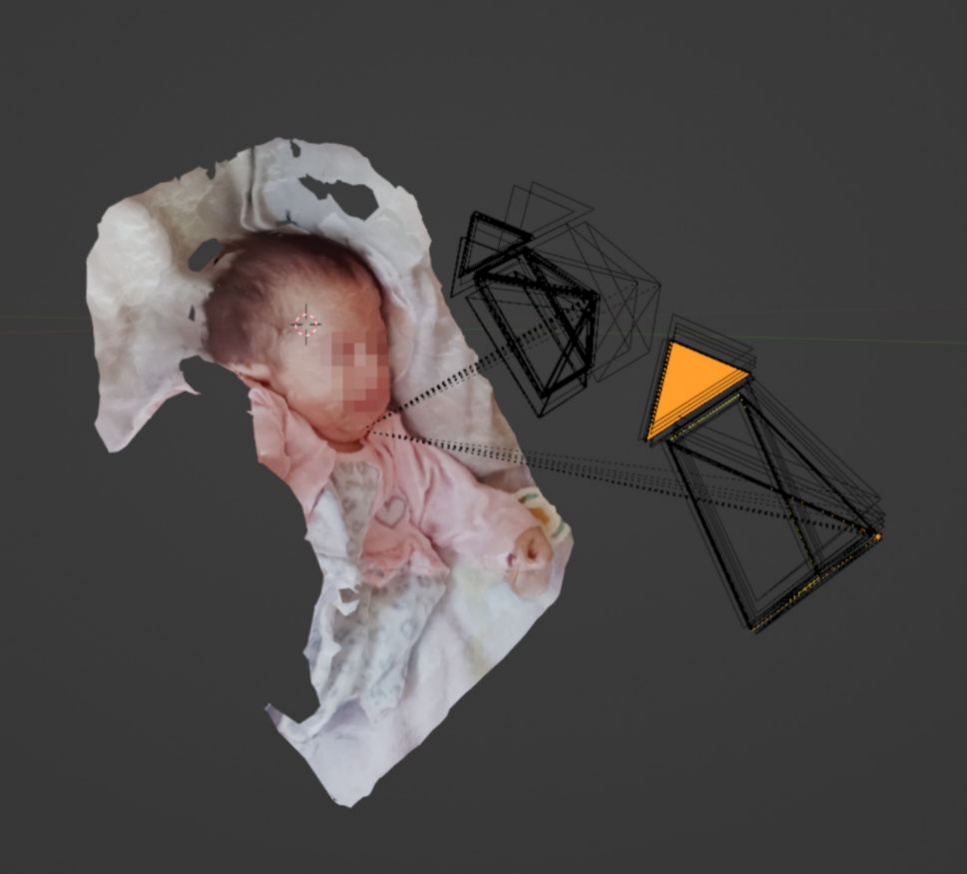
Resulting virtual 3D scene generated with Structure from Motion. The black boxes visualize the determined camera positions of the individual frames at different times of the forensic video. The dotted lines show the viewing direction of the virtual cameras on the reconstructed polygonal 3D mesh of the objects in the scene

The workflow for reconstructing the crime scene with the individual process steps, such as extracting the depth information from the raw material, is shown in Fig. 3. The open source software Meshroom is used for the photogrammetric calculations based on the evidence video recording to create a virtual textured 3D scene. The first step is to split the evidence video into 171 frames using the VLC video player. 121 frames were discarded because of blurring effects [27] and 50 frames could be used for the creation of the virtual scene. It was ensured that the image area of successive frames overlapped by at least 70%, so that the parallax between the images could be determined by photogrammetric calculations. The photogrammetric pipeline in Meshroom basically consists of the steps Feature Extraction [28–30], Feature Matching, Image Matching [31], Structure-from-Motion [32, 33], Depth Map Estimation [33], Meshing (algorithms used based on [33–36]) and Texturing (algorithms used based on [37, 38]). Figure 4 shows the virtual 3D scene resulting from these calculations. The resulting virtual 3D scene is not to scale, it is an arbitrarily scaled scene. Therefore, it must be aligned with a full-scale 3D scan of an object that appears in the evidence video. Here, the 3D scan of the child’s pillow was used as a size reference. Without such an object, the 3D scene cannot be scaled in real scale.

### Real-scale 3D scan of the size reference (Pillow)

**Fig. 5:**
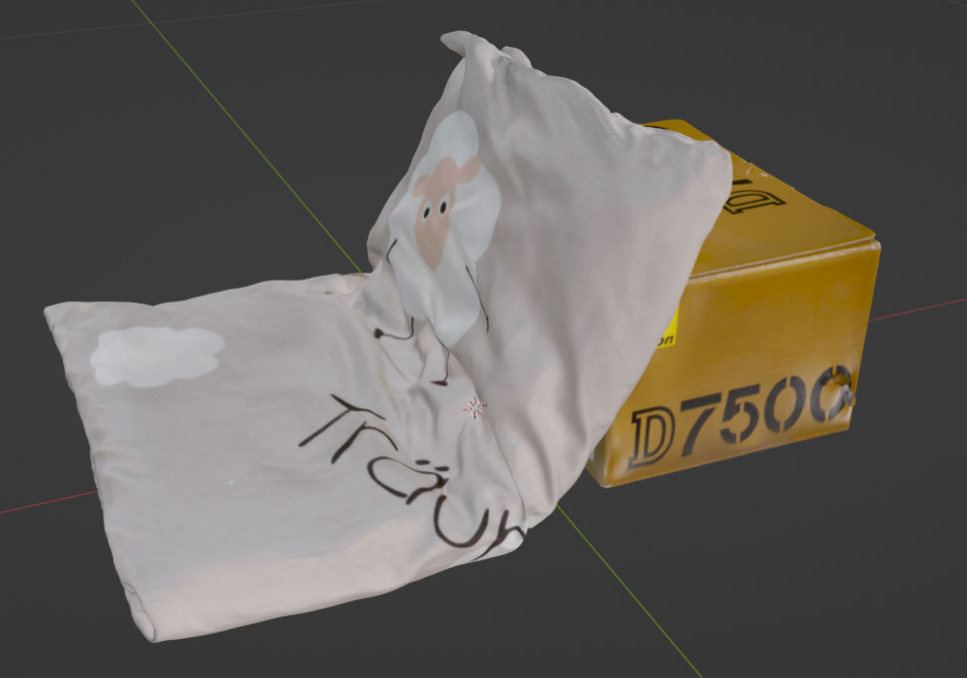
Resulting polygonal 3D mesh from the structural light scan of the cushion. A box was placed under the pillow to approximate the drape from the evidence video. The polygons of the box can be removed in a 3D editing software such as MeshLab (https://www.meshlab.net/)

The Artec Leo structured light scanner is used to create the size reference of the child’s pillow. The pillow is placed on a glass table for the scanning process so that the entire pillow can be scanned without having to rotate it. Figure 2 shows the scan setup. The raw data is imported into the processing software Artec Studio. The individual processing steps in Artec Studio are the Removal of unnecessary Points, Global Registration, Fusion and Texturing. The result after the last step is a textured 3D model of the pillow shown in Fig. 5.

### Superimposition of the size reference with arbitrarily scaled scene

**Fig. 6:**
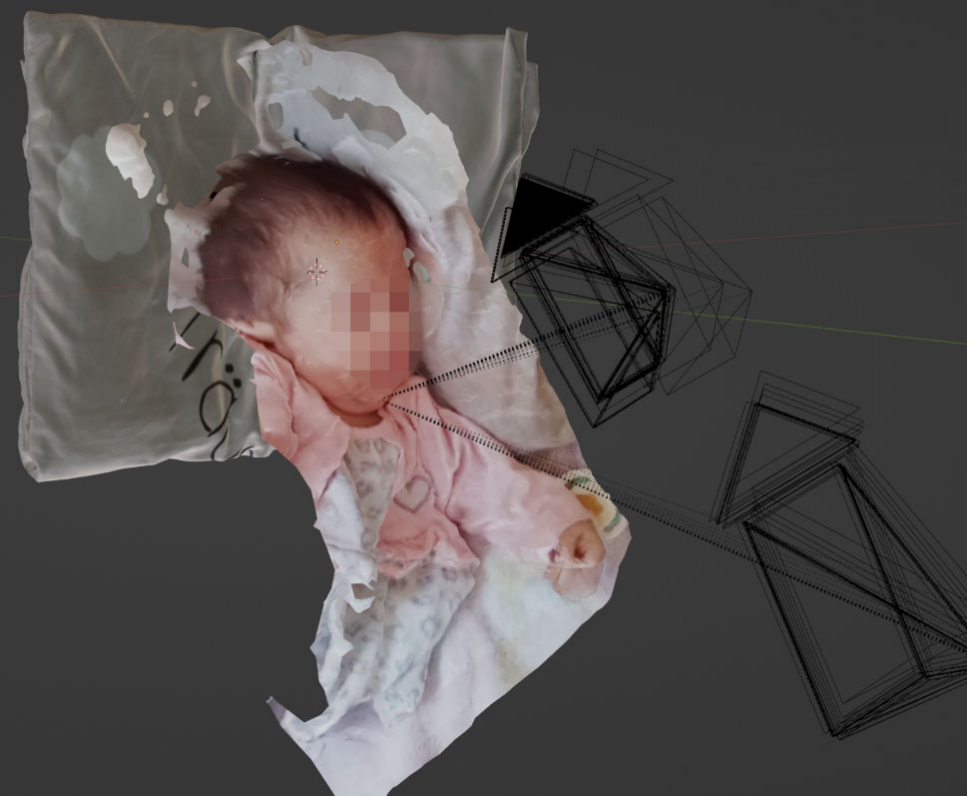
Overlay of the size reference. The arbitrarily scaled virtual 3D scene from Fig. 4 is now aligned with the 3D digital copy of the size reference shown in Fig. 5. Since the 3D scan of the size reference is in real scale, the virtual 3D scene is also in real scale after the alignment. The virtual cameras needed for scaling are shown as black objects to the right of the child. The Fig. 7 below shows a view through such a virtual camera

**Fig. 7:**
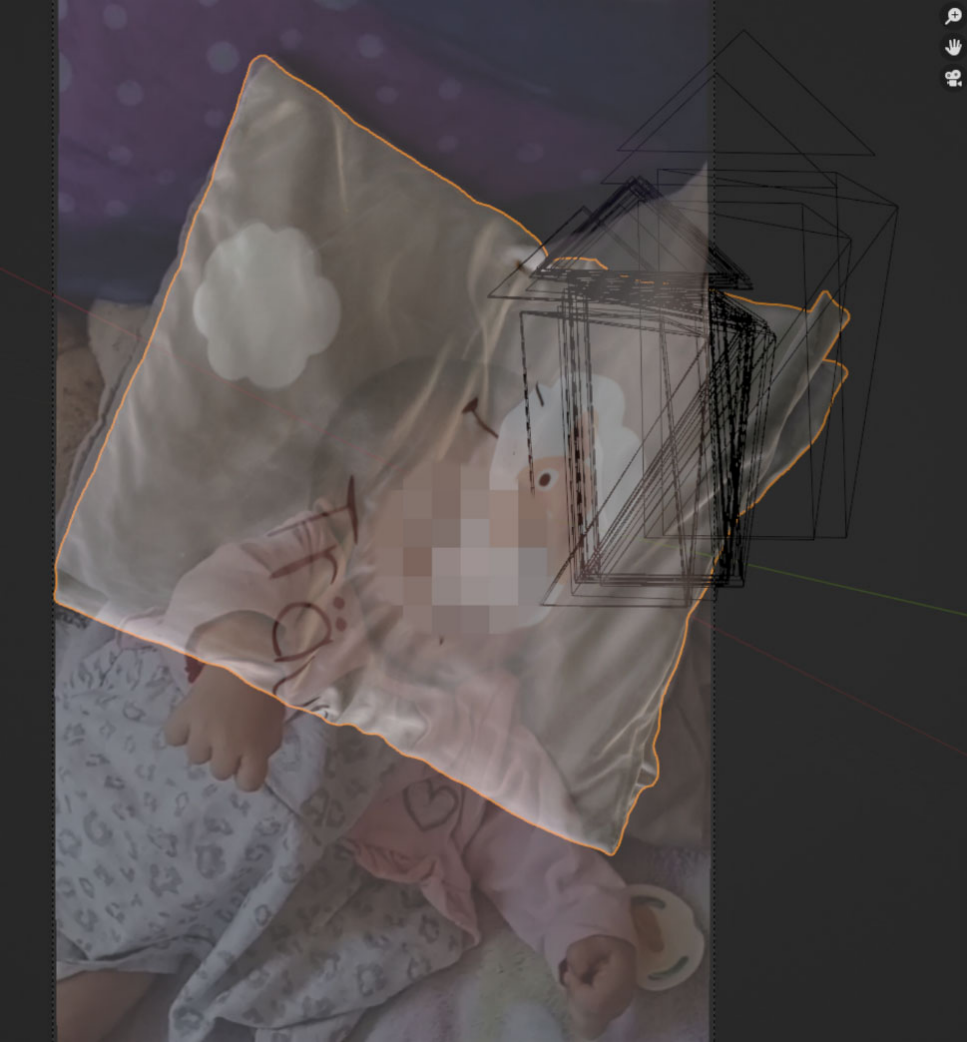
Superimposition of the cloud imprint of the pillow. When the arbitrarily scaled 3D scene is aligned with the size reference, the size reference can be viewed through the virtual camera poses and superimposed on the frames of the evidence video. In this way, the cloud imprint on the 3D scan can be superimposed on the image of the cloud imprint in the evidence video to enrich the virtual 3D scene with the original scale

**Fig. 8:**
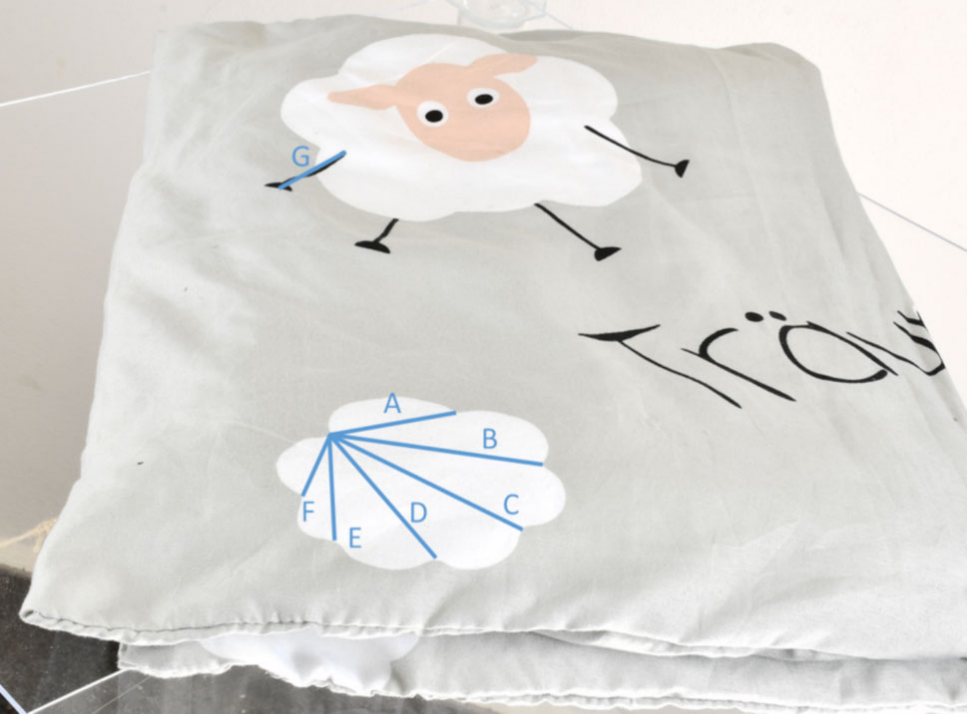
Measurement of the physical cloud imprint, which shall be used as size reference

**Fig. 9:**
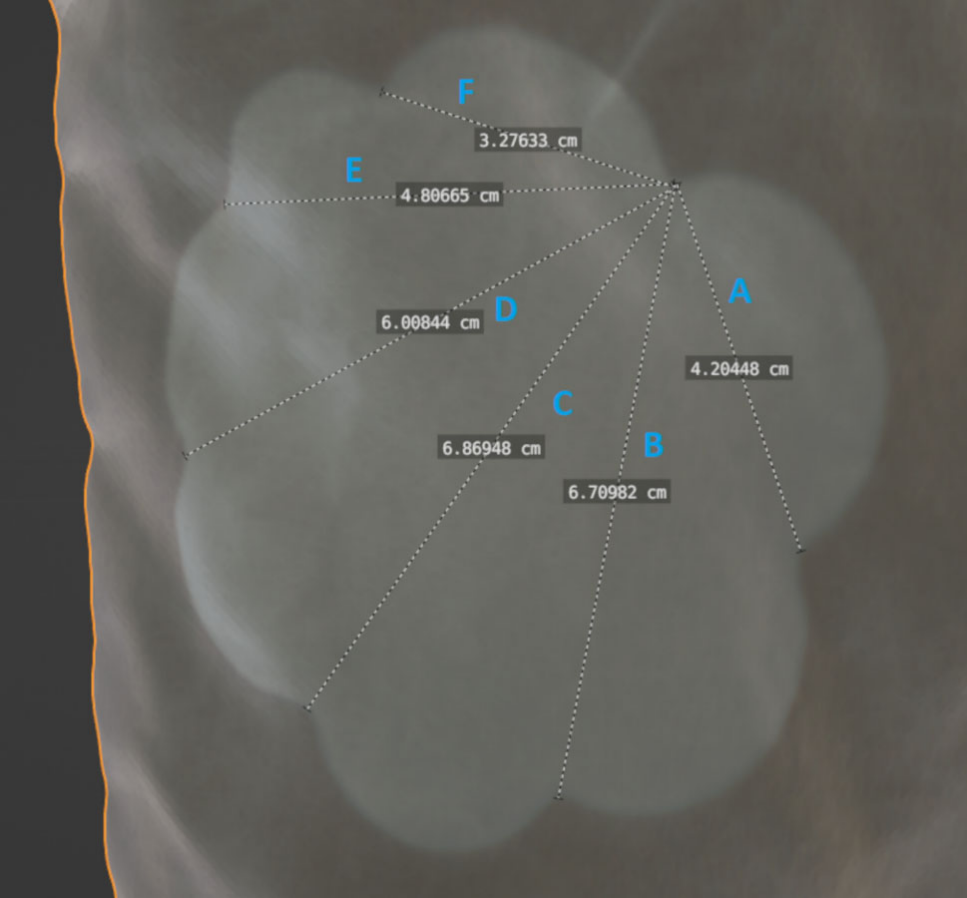
Measurement of the 3D-scanned cloud imprint, which shall be used as size reference. The measured values are shown in Table 1

Superimposition is the alignment of a scene to a size reference. In this case, the arbitrarily scaled scene was aligned to the real scaled size reference of the pillow in the Blender software application. Figure 6 shows the overlay of the arbitrarily scaled scene with the size reference. The overlay can be used to ensure that prominent features of the pillow in the evidence video match the same prominent features on the 3D scanned size reference. Here, a cloud imprint is used as an orientation, which is superimposed on the size reference (see Fig. 7). To verify the scale accuracy of the 3D scan of the pillow, measurements were taken on the physical pillow to compare with the measurements from the 3D scan. Figure 8 shows the physical measurements, Fig. 9 shows the corresponding measurements on the 3D scan of the pillow. This resulted in a maximum absolute deviation of 0.4 cm at measurement distance D. The reasons for the deviations generally shown in Table 1 are the accuracy of the scanning method and the changed folding of the pillow at the time of scanning compared to the time of the physical measurement. The folding of the pillow for the 3D scan was necessary because the shape of the pillow should be reproduced as well as possible, as shown in the evidence video.

**Table 1:** Measurements taken to quantify the accuracy of the reference imprint

Measurement Distance	Measured Value in Scan [cm]	Physical Measurement [cm]	Absolute Difference [cm]
A	4.2	4.2	0.0
B	6.7	6.7	0.0
C	6.9	7.1	0.2
D	6.0	6.4	0.4
E	4.8	5.0	0.2
F	3.3	3.2	0.1

The measuring sections and corresponding measured values are shown in Figs. 8 and 9

### Circumference measurements of the child’s head

**Fig. 10:**
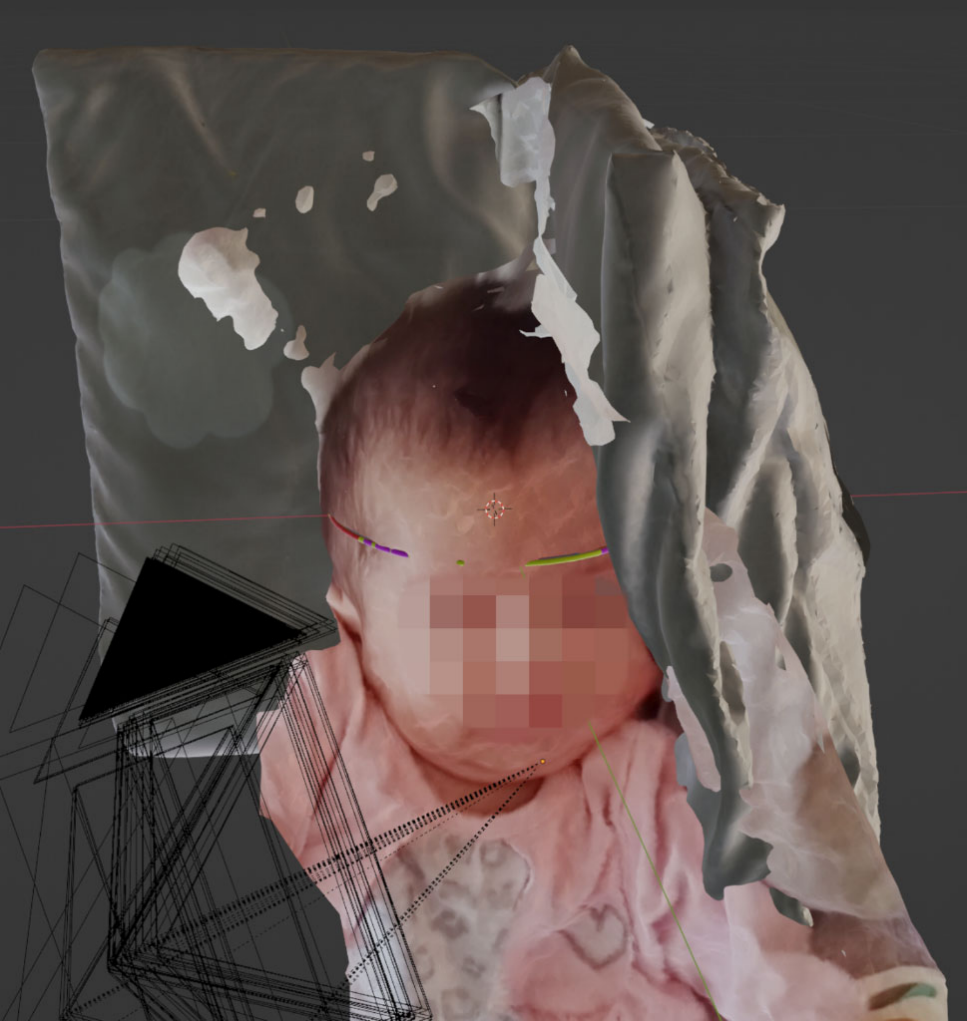
Circumferential measurements as Bézier curves in the virtual 3D scene. The Bézier curves were applied to the child’s head in the now real-scaled virtual 3D scene to output the head circumference. The five measurements that were carried out are shown in different colours. The corresponding values can be found in Table 2

The head circumference is measured by applying a Bézier curve in Blender to the point on the head to be measured. To do this, the head is moved to the origin of the global coordinate system, creating a binding relationship between the entire scene and the head. Consequently, the overlay remains. The global x-axis is set as the symmetry axis of the head, assuming that the left and right sides of the head are symmetrical. A Bézier curve is then created and fitted to the child’s head. The Bézier curve is then converted to a mesh. The curve is now composed of individual edges whose lengths together approximate the measured circumference. A separate B-spline curve was generated for each measurement. A total of five measurements were taken from the child’s head, as shown in Fig. 10.

## Results and discussion

### Results of the head measurements of the child

**Fig. 11:**
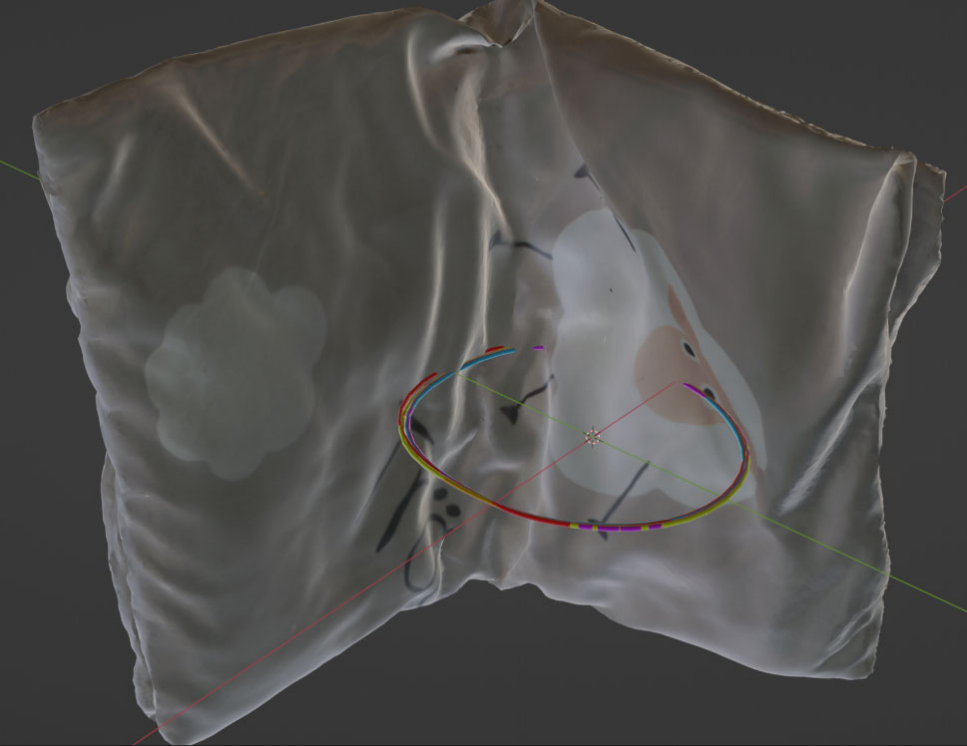
Position of the circumference measurements in relation to the size reference (pillow). This Figure shows the position of the circumference measurements in relation to the pillow. The child’s head has been hidden here. The five measurements that were carried out are shown in different colours. The results of the measurements can be found in Table 2

Table 2 shows the resulting five circumferential measurements of the child’s head in the full-scale virtual 3D scene. The values have a minimum of 43.40 cm and a maximum of 44.38 cm. The standard deviation is 0.39 cm around an arithmetic mean of 43.99 cm and a median of 44.16 cm. Figures 10 and 11 show the location of the Bézier circumference measurements in the virtual 3D scene.

### Discussion of the measurements

The measurements presented in Section 5.1 corroborate with those obtained from the forensic autopsy in the present case, thus reinforcing the hypothesis that an elevated head circumference at the time of recording the smartphone evidence video may be due to external factors. It is important to note that estimation of head circumference is subject to some error, which may lead to the described discrepancy. One potential source of error is the information content of the video, specifically the resolution of the evidentiary video material. In addition, the image area occupied by the relevant objects, image sharpness, blurring and lighting conditions also play a significant role. Further influences can be observed when digitizing the size reference with the Artec Leo Structured Light Handheld Scanner. Transparent, highly reflective or highly homogeneous surfaces are particularly prone to errors during scanning. Structure-from-motion algorithms are used to reconstruct the shape of the object to be measured with Meshroom. In particular, the image resolution, the image noise, the image blur, and the camera angle are of central importance for these algorithms. Furthermore, the number of acquired images has an impact on the quality of the generated 3D scene. During the alignment of the reconstructed 3D scene with the size reference, it is possible that a superposition occurs due to human error resulting from the transformation tools in Blender. To perform a circumference measurement using Bźier curves, it is necessary to insert the evidence video into the overlay within the 3D scene. This insertion depends on the overlay and thus on the perspectives of the 3D scene captured by the camera movement. For those parts of the scene that were not captured in the evidence video, such as the back of the head, assumptions must be made. In this case, the right half of the head was assumed to be symmetrical.

Regarding the accuracy and reliability of reconstructions based on low-quality video material and the associated measurement uncertainties, the following can be added The exact quantitative determination of the measurement accuracy requires a rigorous comparison between the reconstructed 3D models and known physical measurements. This methodology has been meticulously applied in the master thesis of Hanjo Tim Fritzsch using the workflow presented in this paper. To ensure robustness, multiple reconstructions of the same scene were generated to provide a comprehensive data set to assess both the variability and accuracy of the measurements. The collected data were subjected to statistical analysis to quantify the reconstruction error (SRe) and its deviation from the physical reference values. The reconstruction error (SRe) quantifies the deviation of the unknown arithmetic mean ($\upmu$i) of the reconstruction trials from the reference value ($\upmu$). For the Bźier perimeter measurement, the maximum a posteriori estimate of the SRe is 1.505442 mm, with a 90% interval ranging from 0.0000586 mm to 9.607803 mm. This metric is essential to determine the overall accuracy of the reconstruction process and to identify potential discrepancies between the reconstructed model and the actual physical measurements. The intraobserver error (IOE) indicates the dispersion of individual measurements within a single reconstruction trial around their unknown mean ($\upmu$i). For the Bźier perimeter measurement, the IOE lies between 0.8864607 mm and 2.3638025 mm with 90% confidence, with a maximum a posteriori estimate of 1.652649 mm. This type of error is crucial for assessing the consistency of measurements made by the same observer under identical conditions. It should be noted, however, that in this case there were no reference measurement sections available, only areas of the pillow (the cloud) that had already been analyzed. However, the recording conditions of the master’s thesis can be applied to the present case. This is especially true for determining whether the head circumference in the private photographs was unnaturally inflated or not. Even taking into account the error tolerances and applying them to the present case, unnatural head circumferences result and offer support in categorizing if the child has been abused.

**Table 2:** Measurements of the child’s head circumference in relation to Figs. 10 and 11

Color coding in Fig. 11	Circumference [cm]
red	44.16
orange	44.38
blue	43.40
green	44.34
pink	43.67

### Integration into the current state of research

The integration of two-dimensional data, such as photographs and video, with three-dimensional models adds value to the documentation and analysis of forensic issues. In addition, it is possible to supplement standard procedures in forensic science and legal medicine, such as the interpretation of injuries. This complementation is provided by the present work. Similar approaches are described by Massini et al. [12], who extend 2D photographs with 3D documentation technologies with the aim of better interpreting wounds. The measurements presented in this real case study are circumferential measurements. Comparable procedures for object measurements are presented by Villa [10], where hand injuries were measured in two and three dimensions. In the present work, a pillow was digitized using a structured light scanner and used as a reference measurement for further analysis. Similar goals were pursued by Urschler et al. [39], who considered different technologies to create 3D models, especially for reference scans. The research group of Koller et al. [40] analyzed the measurement of 3D objects in their study, once with VR and once with on-screen measurements. The researchers found that discrepancies, for example in the maximum length of a wound, averaged 2.7 mm between the model and the reference. Similar results were found by Michienzi et al. [41], who reported average discrepancies of 2.3 mm between the model and the reference. These discrepancies were found in models created using photogrammetric methods. The use of laser scanners makes it possible to achieve higher scanning accuracy, as demonstrated by Holowko et al. [13] in their study. In this study, 3D models of walls were generated by utilizing close-up scans with an accuracy of up to 0.25 mm. The present study also yielded comparable results. The absolute difference between the 3D model and the analog pillow was 4.0 mm (all values are shown in Table 1). In addition to the technical accuracy of the scanning system, the documented discrepancies are attributed to the different folding of the pillow. The mean head circumference measurement was 43.99 cm (all values can be found in Table 2), with a reference value of 44 cm. Similar results were obtained in the study by Sieberth et al. [19] for limb measurements. The precision achieved in the creation of three-dimensional scenes with the presented method allows the measurement of objects within the scene in a way that can support forensic and medico-legal investigations.

## Conclusion and further fields of application

### Conclusion

The presented workflow can help to estimate the size of objects in videos. Possible applications would be, for example, in cadaver examinations to determine the circumference of body parts (in addition to the head, as in the present case) in order to draw conclusions about possible causes of injury or death. Furthermore, concrete questions could be answered in the analysis of objects. Here, the presented Bézier circumference measurement method could be used to measure the circumference and size of objects in order to compare them with suspicious or known patterns. An advantage of the workflow is that very little information about the physical scene other than the video is needed to perform the reconstruction and measurements. For example, the reconstruction in the present case required only a 10-second evidence video clip and the secured pillow as a physical size reference. Using the presented method, it was possible to measure the child’s head circumference in the underlying smartphone evidence video. Based on the results, it can be assumed that the child had already been abused at the time of the video recording and that the head circumference had not grown naturally. However, it is important to note that this is not a definitive statement about actual injuries and that medical experts, such as forensic pathologists, must interpret these results.

In principle, the use of these technologies is possible in various forensic cases, provided that video material is available and meets certain requirements. These include the presence of sufficient image characteristics. Examples include the resolution or number of pixels representing the object of interest in the image, the exposure time, and general camera parameters [42, 43]. In addition, a parallax shift is required to compute a 3D structure using appropriate algorithms [44]. A major challenge is the availability of a reference object to scale 3D scenes based on photogrammetric reconstruction methods. These are some of the limitations and challenges that can arise in the daily forensic analysis of image and video material. The presented workflow has a high potential in this field, but could also be used to support other investigations.

### Further fields of application

Forensic 3D reconstruction involves meticulously recreating crime scenes and synchronizing them with surveillance camera footage. Typically, detailed 3D scans of the crime scene are created and enriched with additional case-specific information, such as 3D animations. This technique allows for a comprehensive visualization of the spatial and temporal relationships between the crime, the people involved, and the location [45]. However, there are cases where the exact crime scene is either unknown or inaccessible for direct scanning. In such cases, 3D scene reconstruction from video footage can be an effective alternative to acquire and visualize critical information about the crime scene. Unlike traditional 2D image processing techniques, 3D reconstruction offers significant advantages; it not only provides accurate measurements, but also allows the virtual environment to be used in further 3D simulations and animations [13]. In addition, the virtual scene can be integrated into immersive experiences through virtual reality (VR) or augmented reality (AR) technologies, enhancing the depth and utility of forensic analysis [46]. Such advanced applications demonstrate the potential of 3D reconstruction to transform forensic investigations by providing dynamic and interactive ways to analyze and present crime scene data. This approach not only aids the investigative process, but also serves as a powerful tool for courtroom presentations, providing judges and juries with a clear and detailed understanding of the crime scene.

## Key Points

This case involves possible child abuse of an infant. The cause of death was found to be a chronic progressive subdural hematoma combined with an abnormally swollen head.

The only evidence that could have clarified the time of possible child abuse was a private video of the child’s parents showing the child lying on a pillow.

The purpose of the procedure was to prove that the child’s head was already unnaturally swollen in this video. The video itself and the pillow on which the child was lying on in the video were used for the analysis.

3D shape extraction methods were used to create 3D models from the available video and the child’s pillow. With the help of superimposition methods the 3D models were scaled for circumference measurements on the child’s head.

Based on the head circumference measurements of the child in the created 3D scene, it can be assumed that the child had already been abused at the time of the private video recording.
